# Studies on Molecular Dynamics of Intrinsically Disordered Proteins and Their Fuzzy Complexes: A Mini-Review

**DOI:** 10.1016/j.csbj.2019.06.009

**Published:** 2019-06-13

**Authors:** Kota Kasahara, Hiroki Terazawa, Takuya Takahashi, Junichi Higo

**Affiliations:** a College of Life Sciences, Ritsumeikan University, 1-1-1 Noji-higashi, Kusatsu, Shiga 525-8577, Japan; b Graduate School of Life Sciences, Ritsumeikan University, 1-1-1 Noji-higashi, Kusatsu, Shiga 525-8577, Japan; c Graduate School of Simulation Studies, University of Hyogo, 7-1-28 Minatojima-minamimachi, Chuo-ku, Kobe 650-0047, Japan

## Abstract

The molecular dynamics (MD) method is a promising approach toward elucidating the molecular mechanisms of intrinsically disordered regions (IDRs) of proteins and their *fuzzy complexes*. This mini-review introduces recent studies that apply MD simulations to investigate the molecular recognition of IDRs. Firstly, methodological issues by which MD simulations treat IDRs, such as developing force fields, treating periodic boundary conditions, and enhanced sampling approaches, are discussed. Then, several examples of the applications of MD to investigate molecular interactions of IDRs in terms of the two kinds of complex formations; c*oupled-folding and binding* and fuzzy complex. MD simulations provide insight into the molecular mechanisms of these binding processes by sampling conformational ensembles of flexible IDRs. In particular, we focused on all-atom explicit-solvent MD simulations except for studies of higher-order assembly of IDRs. Recent advances in MD methods, and computational power make it possible to dissect the molecular details of realistic molecular systems involving the dynamic behavior of IDRs.

## 1 Introduction

Within the past several decades, protein science has focused primarily on the correlation among sequence, structure, and function of proteins based on Anfinsen's dogma, stating that the amino acid sequence of a protein encodes its three-dimensional (3D) structure [1]. Elucidation of the principles of this relationship has been a long-standing conundrum in this field of study. [2,3] To help resolve this issue, extensive efforts have been made in structural biology to reveal the 3D structures of a variety of proteins to establish a 3D structure database. [4] Approaches using structural bioinformatics have used this compiled data to begin to elucidate the sequence–structure–function paradigm based on a data-driven approach [5,6]. However, the current knowledge of protein structures is biased toward globular proteins. It is difficult to analyze highly complex molecules and their assemblies (e.g., supermolecular complexes and membrane proteins). Although recent advances in electron microscopy and hybrid approaches are promising toward resolving this issue [7], characterizing the structural details of the protein regions that have extreme flexibility is not straightforward [8]. Such a flexible region is termed “intrinsically disordered region” (IDR), and a protein including IDRs is termed “intrinsically disordered protein” (IDP).

The biological importance of IDRs has been widely accepted. Several studies have been conducted to reveal the nature of IDRs, and several review articles have been published [9,10]. However, the molecular mechanisms of IDRs remain largely undisclosed. A protein that conducts its molecular functions without having a particular conformation appears to be inconsistent with the sequence–structure–function paradigm. “Coupled-folding and binding” is a typical protein conformation scenario in which a stable protein conformation is formed only when IDRs are specifically recognized by a receptor [8]. In this mechanism, IDRs only partly conform to the sequence–structure–function paradigm because their functions are conducted with their specific structures in the bound state. However, there are many exceptions to this, indicating that IDRs do not always take on a specific conformation, even after binding with their receptors. Complex formations without a specific 3D complex structure, a “fuzzy complex,” have been reported [11,12]. This term covers a wide spectrum of binding mechanisms from “polymorphic” to “random”. A polymorphic fuzzy complex indicates that IDR binds to its target using some distinct binding modes separated by low-energy barriers that can be easily exceeded within the equilibrium. A random fuzzy complex indicates that IDR dynamically fluctuates without any particular binding conformation but continues to interact specifically to its receptor.

To understand the principles by which IDRs recognize these these receptors, structural information at the atomic level is of importance. However, their extreme flexibility makes it difficult to characterize the conformational ensembles of IDRs at the atomic level. In addition to a variety of experimental techniques, such as circular dichroism (CD), nuclear magnetic resonance (NMR), small-angle X-ray scattering (SAXS), and fluorescence resonance energy transfer (FRET), the molecular dynamics (MD) method provides a great insight [[13], [14], [15]]. The MD method simulates the evolution in time of a given molecular system described as a set of atomic coordinates (and velocities) in Cartesian space. Although this method requires considerable computational costs, recent advances in computational power, algorithms, and methods have made it possible to analyze large molecular systems over long time scales.

In this mini-review, we introduce recent advances in MD studies on IDRs and their fuzzy complexes. We discuss the methodological advances and current problems of the MD method for simulating IDRs in the section “Methods for IDR simulations”. Then, the sections “Coupled-folding and binding” and “Fuzzy complexes” present recent applications of the MD method to dissect the molecular mechanisms of IDRs.

## 2 Methods for IDR Simulations

### 2.1 Force Fields

Results of MD simulations depend highly on empirical parameters for potential energy functions, or force fields. Therefore, an adjustment of the parameters is a long-standing issue. A typical problem in simulating structured proteins is adjusting the stabilities of secondary structural elements. Recent important advances in computational power have enabled the exploration of a wider range of the parameter space and extensive validations. Several state-of-the-art force fields can yield results that quantitatively agree with various types of experimental results for structured proteins [16]. However, many force fields are parameterized to fit the properties of structured proteins and are not suitable to apply to IDRs. It is known that these force fields tend to overstabilize compact forms of proteins, as compared with experimentally measured characteristics. In some force fields, radius of gyration (*R*_g_) of an unstructured protein is underestimated, as compared with experimental values measured using FRET and SAXS [[17], [18], [19], [20], [21]]. Comparisons using hydrogen-exchange experiments suggest overstabilizing intramolecular hydrogen bonds in proteins [19]. Behavior of unfolded peptides in MD simulations depends strongly on the force field and water models. [16,22].

One typical strategy used to adjust the main chain conformation is to tune the potential parameters for main-chain dihedral angles. The AMBER ff14IDPSFF force field revised the standard AMBER ff14 force field for dihedral potentials [23]. The CHARMM36m force field was developed to revise the CHARMM36 force field [24]. The potential of guanidium group to pair with carboxylate group was weakened in addition to adjusting the main-chain dihedral potentials. As other approaches, some studies focused on protein–water interactions. Best et al. [17] have proposed a modified version of the AMBER ff03 force field, AMBER ff03ws, strengthening the Lennard-Jones (LJ) potential for the protein–water interactions. Although AMBER force fields apply the Lorentz–Berthelot combination rule to calculate atom interactions with different atom types, AMBER ff03ws introduces an exception to this rule by applying a scaling factor for the protein–water interactions. Alternatively, a new water model, TIP4P-D, was developed by deepening the energy well in the LJ potential function of the oxygen atom of TIP4P [20]. These two approaches that focused on protein–water interactions resolved the issue of underestimating *R*_g_. Henriques and Skepö [25] have confirmed that SAXS profiles of histatin 5 protein are well reproduced under both AMBER ff03ws with TIP4P/2005 and AMBER ff99SB-ILDN with TIP4P-D. Nerenberg et al. [26] have applied a similar approach for optimizing solute–water interactions to improve the reproducibility of solvation free energy for small compounds. On the other hand, it has been reported that a different approach using a force field based on the Kirkwood–Buff theory also resolved the issue of overcompaction of IDR conformation in simulations [27].

Although these new force fields improved the reproducibility for the experimentally measured conformational properties of IDRs, some drawbacks have been reported. The benchmark using the RS peptide, reported by Rauscher et al. [28], presented an inconsistency with experimental observations in some of the state-of-the-art IDP-oriented force fields. The use of CHARMM22*, which is not tailored to IDR simulations, with the TIP3P water model yielded a better ensemble than a combination of AMBER ff03ws and TIP4P-D. Robustelli et al. [29] have recently reported a comprehensive benchmark of 6 IDP-oriented force fields with 21 proteins, both structured and disordered proteins. These currently proposed IDP-oriented force fields failed to reproduce conformational ensembles of structured proteins. Based on the benchmark, they have proposed a new force field, AMBER ff99SB-disp, by adjusting the parameters of the backbone dihedral, LJ potentials for carbonyl oxygen and amide hydrogen pairs and side-chain dihedral. In addition, modified version of TIP4P-D water model also presented. They have reported that this new force field yielded better consistency with experimental observations for a variety of proteins than the other force fields examined in the benchmark.

These recent studies in force field development imply that an accurate description of hydration properties is a key feature. Rani et al. [30,31] have presented the differences in hydration properties between structured protein and IDPs. Although MD simulations have been successful in analyzing the thermodynamic properties of hydration, dissecting the dynamic and kinetic properties of water molecules around an ionic solute is not as straightforward, even for simple molecular systems, such as solvation of a monovalent atomic ion. [32,33] The results of our previous study have suggested that none of the 10 standard water models for the classical MD simulation reproduced experimentally measured water diffusion coefficients and rotational relaxation times [34]. Ding et al. [35] have suggested that quantum mechanics is necessary to reproduce anomalous diffusion of water molecules around an ionic solute. Thus, classical models for an accurate description of water hydration must be invented.

### 2.2 Long-Range Electrostatic Potential and Periodic Boundary Condition

The other key factor for calculated potential energy is the electrostatic potential. The de facto standard approach is the Ewald-based method, which assumes a periodic boundary condition (PBC) and calculates the electrostatic potential with Fourier transform [36]. It is argued that this approach produces some artifacts that originate from PBC considering an infinite array of molecules with identical configurations. Although these artifacts have been examined, the approach remains controversial because contradicting conclusions about whether the artifact is significant, have been reported [37,38]. This discrepancy might be caused by using insufficient samples [39]. Our previous study examined the finite-size effects under Ewald PBC conditions using an enhanced sampling method, the cumulative trajectory length of which was 192 μs [40]. Consequently, the ensembles having smaller PBC cells showed extended conformations that were slightly more stable, although the overall geometry of the free-energy landscapes were similar. However, these studies were conducted using simple model peptides, such as alanine octapeptide. The significance of these types of artifacts for IDRs remains unclear. In general, because IDRs tend to have many charged residues, the impacts of artifacts in electrostatic potentials are expected to be higher than those from peptides consisting of nonpolar residues. These differences would be examined in future studies.

To resolve the artifact issue, non-Ewald methods have been extensively developed [41]. A major concern in using non-Ewald methods is that artifacts arise from the cutoff truncation of long-range electrostatic potential. It has been reported that use of the non-Ewald methods for unstructured polypeptides tends to yield smaller *R*_g_ and more flexible conformations than the Ewald-based methods [21,42]. Our group examined a non-Ewald method, the zero-multipole summation method (ZMM) [43,44], which was inspired by the findings of Wolf et al. [45], which have suggested that artifacts caused by cutoff truncation are minimized when the net charge in the cutoff sphere is zero. ZMM is formulated to cancel summation of multipoles in the cutoff sphere. We demonstrated a high consistency between free-energy landscapes calculated from the particle-mesh Ewald method and ZMM when the simulation cell is sufficiently large. [40] Additionally, ZMM resulted in smaller discrepancies among the simulations using different sizes of cells, as compared with the use of an Ewald-based method.

### 2.3 Conformational Sampling

In addition to the accuracy of potential calculations in each step, methodology to explore the conformational space of a molecular system is also an important issue for efficient simulation. Because IDRs have extremely high conformational diversity, high computational costs are required to explore their large conformational spaces. In particular, the molecular system is, at times, trapped in minor basins within the free-energy landscape and should overcome energy barriers to be able to globally explore the conformational space. One simple approach is running a large number of canonical MD simulations with diverse initial conditions. Although this approach can easily be applied using any MD simulation package, resultant ensembles are highly dependent on the initial conformation of each simulation. Obtaining an accurate canonical ensemble of an IDR with tens of residues may require impractically long simulations. To resolve this issue, enhanced sampling methods, such as multicanonical MD (McMD) [46] and replica-exchange MD (REMD) [47], are promising, These methods apply artificial potential or forces to overcome the energy barriers. A variety of enhanced sampling methods have been extensively developed and examined. More details about this issue have been discussed in our previous reviews [48,49]. In general, these enhanced sampling methods involve some technical issues such as fine tuning of adjustable parameters. For example, the resultant ensemble of a REMD simulation depends on the setting of the total length of trajectory, the number of replicas, and interval times for replica-exchange trials [[50], [51], [52], [53], [54], [55]]. Because there is no gold standard for adjusting these parameters, techniques and experiences to setup the simulation conditions are required for users.

## 3 Coupled-Folding and Binding

Taking advantages of extensive developments of the methodologies described above, the MD method has been widely applied to investigate molecular mechanisms of IDRs. A major question is the atomic details of the coupled-folding and binding phenomena. During the process of coupled-folding and binding, an IDR forms a specific conformation by binding to its receptor. Although many 3D structures of IDRs in the bound state have been identified, the details of the molecular mechanisms of the disorder–order transition remain unclear. For this, two well-known models—induced folding (or induced fit) [56] and conformational selection [57]—have been presented. The former indicates that the bound conformation of an IDR is induced by interactions with a receptor. The latter indicates that the conformational ensemble of an isolated IDR includes its bound conformation, and that this conformation is then populated by binding to a receptor. As described in this subsection, several studies suggest that coupled-folding and binding processes follow a mixed scenario of these two models. For example, the bound conformation of an IDR is partially preformed in the unbound state, and folding of the entire binding region is induced by binding. Alternatively, binding process follows multiple pathways with both the models. Conformational ensembles of an IDR in unbound, bound, and transition states obtained by all-atom MD simulations provide insight into the spectrum of coupled-folding and binding scenario.

### 3.1 Conformational Ensembles of Unbound IDRs

Conformational ensemble of an unbound IDR provides great insight into the coupled-folding and binding mechanism. If an unbound IDR does not form folded conformation similar to its bound state, the folding would be induced by binding (induced fit model). For example, Mittal et al. [58] reported their results of REMD simulations for an unbound 15-residue segment in the N-terminal IDR of transcription activation domain of p53, which is a ligand of MDM2. Although the IDR dynamically fluctuates and frequently interconverts among a variety of conformations that are separated by low energy barriers, the central region of the 15-residue segment exhibited high helix propensity, which is similar to the bound state. Knott and Best [59] also reported that an IDR of NCBD, which is a ligand of ATCR, partially preformed a secondary structure in the unbound state by using all-atom REMD simulations. Bernetti et al. [60] showed that unbound Sev N_TAIL_ rapidly interconverts among several substates separated by low energy barriers by using metadynamics and a kinetic Monte Carlo approach.

In many cases, an unbound IDR has a certain secondary structure propensity to form a conformation similar to its bound state. This preformed structural element usually reproduces a part of the bound conformation and might help in the recognition of its receptor [61].

### 3.2 Conformational Ensemble of Bound IDRs

To capture the conformational ensemble of an IDR in the bound state, MD simulations must be performed with a molecular model including both the ligand and receptor. Although the computational cost increases with an increase in the model size, a decrease in flexibility binding makes conformational sampling easier. Wostenberg et al. [62] reported that the C-terminal IDR of FCP1 dynamically fluctuates even in the complex with RAP74. For a wider conformational space including bound–unbound equilibrium applying the enhanced sampling method is promising approach. The first example is McMD simulations of a short IDR of NRSF with and without its receptor, mSin3 [63]. According to the 3D structure data identified using NMR, a short IDR of NRSF is buried in and recognized by the groove of the paired amphipathic helix domain of mSin3 [64]. The simulation of the dimer system began with a random conformation of a truncated IDR of NRSF located far from the binding site. Consequently, the conformational ensemble of isolated NRSF included a conformation that was similar to that of its bound state. The interactions with mSin3 drastically changed the free-energy landscape of NRSF and guided its conformational changes to the bound state. The free-energy landscape implies that a mixed mechanism of both conformational selection and induced folding occurs for the actual binding processes. On the other hand, the conformational diversity of NRSF was increased by interactions with mSin3. Although the unique bound conformation is highly stable, several minor conformations were also observed. Most conformations of the NRSF peptide in the isolated state have also been observed in the bound state.

Recently, several studies applying enhanced sampling methods with all-atom explicit-solvent models have been reported. Umezawa et al. [65] applied the McMD approach to pKID–KIX complex. They reported that the N-terminal half of the disordered pKID exhibited a strong helix propensity, and the C-terminal half tended to be folded into a helix after binding. Ithuralde et al. [66], investigated the c-myb–KIX complex by using umbrella sampling. Multiple pathways of coupled-folding and binding were reported. Han et al. [67], combined the REMD and metadynamics methods to explore the conformational space of the α-MoRE-MeV–XD complex. They reported that complex formation induced folding of α-MoRE-MeV.

To sample the conformational ensemble focusing on the transition state, Karlsson et al. [68] applied experimentally observed Ф_b_ values to narrow the conformational space to be explored. They reported the conformational diversity of the transition state of the coupled-folding and binding process of the ACTR–NCBD complex. The simulated annealing method was applied with some restrictions defined from the experimental values. Although the structurally heterogeneous ensemble of the transition states implies an induced fit scenario, which also includes secondary structural elements similar to the bound state, suggesting that conformational selection also works partially.

### 3.3 The C-Terminal Domain of the Transcription Factor p53

As shown above, coupled-folding and binding processes have been extensively studied for various targets by using all-atom explicit-solvent MD simulations. One of the, representative example is a short segment in the C-terminal domain of the transcription factor p53 (p53-CTD). This IDR segment has different receptors with distinct conformations, and the receptor specificity is modulated by acetylation of the segment's lysine (Lys) residue. The coupled-folding and binding processes of this hub-like p53-CTD have been extensively investigated. The conventional canonical MD methods have revealed the submicro or micro second–order dynamics of the p53-CTD peptide and its receptors beginning with their experimentally determined structures [[69], [70], [71]]. To explore a wider range of conformational space and to exceed the limit of the time scale, the generalized-ensemble approach has been applied [69,72,73]. Because this approach requires high computational costs, implicit solvent models have been used in these studies. Our group reported on the conformational ensembles of the isolated p53-CTD peptide with/without acetylation by the McMD method using an explicit solvent model [74]. Additionally, a study on the complex formation with S100B using the same method has also been reported [75]. This simulation, which began from a random coil conformation in p53-CTD located far from the binding site in S100B, has suggested a conformational ensemble of p53-CTD around S100B, including a p53-CTD helical bound conformation similar to the structure identified using NMR. These previous simulation studies on p53-CTD have presented that the conformational ensemble of the isolated p53-CTD includes conformations in bound states. In other words, the bound conformations are preformed in an isolated state. Additionally, they suggest that the formation of the encounter complex is driven by long-range interactions and fly casting–like mechanisms, and that the bound conformations are then created by short-range interactions. The fly-casting mechanism is an important feature of the IDR for the formation of encounter complex. High flexibility and a high *R*_g_ increase the chances of the IDR having contact with other molecules in the environment. This enables the IDR to recognize its specific receptor. Early contact between IDR in a random conformation and its receptor induces IDR conformational changes into a specific bound state. These features play pivotal roles in p53-CTD's hub-like property.

## 4 Fuzzy Complexes

In contrast to coupled-folding and binding, some types of IDRs can bind to their receptor without creating a unique stable conformation. Such binding phenomena can roughly be dividedinto two classes: binding between an IDR and an ordered region and binding among multiple IDRs. In any case, the characterization of the conformational ensembles of such fuzzy complexes is not straightforward because of the huge volume of the conformational space that must be explored.

### 4.1 Multimodal Interactions Between an IDR and Ordered Domain

**Fig. 1:**
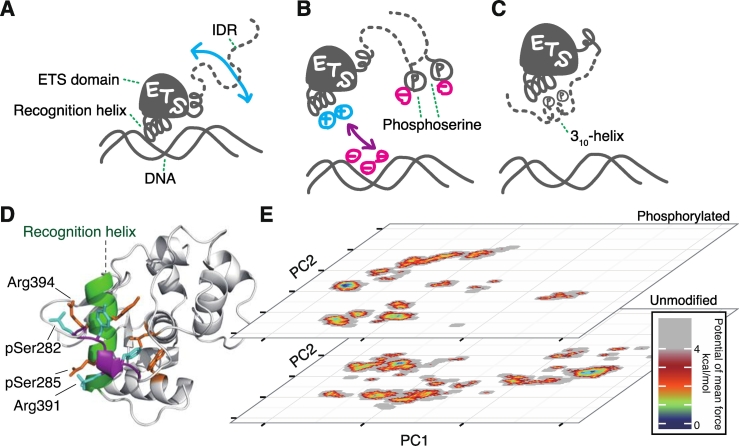
Multimodal interactions between the intrinsically disordered regions (IDRs) and the ETS domain. (A, B, C) Schematic illustrations of the interactions. (A) Ets1 recognizes DNA with the recognition helix. IDR is separated from the DNA-binding region. (B) Phosphorylation of multiple serine (Ser) residues in IDR inhibits DNA binding. (C) A representative of the multimodal (or polymorphic) interactions between IDR and the ETS domain. Phosphoserine residues form salt bridges with basic residues in the recognition helix. (D) Snapshot from our simulation of the same state as that in panel (C). The recognition helix is shown in green. The Ser-rich region with two phosphoserine residues is shown in magenta. (E) Free-energy landscapes of IDR in phosphorylated and unmodified states. The horizontal and vertical axes indicate the first and second principal component axes (PC1 and PC2), respectively. The graphics in panels (D) and (E) are reprinted from Ref. [79] with some modifications under the Creative Commons license.

In the former case, which is the order–disorder binding, IDR typically works as a regulator for the binding target. One prominent example of this is the transcription factor Ets1, which plays important roles in cancer development and autoimmunity [76]. The core domain of Ets1, the ETS domain, recognizes an enhancer region in DNA, and this binding activity is regulated by the IDR neighboring this domain (Fig. 1A). Several phosphorylation sites are localized in this IDR (serine [Ser]-rich region), and the phosphorylation triggers autoinhibition of DNA-binding activity (Fig. 1B). The atomic coordinates of the phosphorylation sites have not been distinguished in the crystal structure of the phosphorylated Ets-1 [77]. Although the previous NMR measurements detected interactions between the phosphorylated IDR and ETS domain [78], the details of the molecular mechanisms have not been clarified.

The MD method has been widely applied for elucidating the molecular mechanisms of autoinhibition of Ets1. Karolak et al. [80] have analyzed the stability of the inhibitory helix unfolding when Ets1 recognizes DNA using replica-exchange MD. The allosteric network of the molecular assembly centered on Ets1 was analyzed based on the fluctuation of the molecules observed in the canonical MD simulations [[81], [82], [83]]. To observe the mechanisms of IDR phosphorylation, we conducted McMD simulations of an Ets1 construct composed of the DNA-binding domain and part of IDR consisting of 25 amino acids (Fig. 1D) [79]. The conformational ensembles of IDR were analyzed in both the unmodified and phosphorylated states. The resultant free-energy landscapes indicated multimodal interactions between IDR and the DNA-binding domain. The two landscapes showed clearly distinct distributions. The phosphorylated IDR tended to interact with the DNA-binding domain, and the most stable conformation exhibited a bifurcated salt bridge with two phosphoserine residues and two acidic residues in the DNA recognition helix (Fig. 1C, D). This mode of interaction can competitively inhibit DNA binding, consistent with experimental observations. Interestingly, the region around these two phosphoserine residues exhibited a helical propensity. In particular, this formed 3_10_-helix in the inhibitory form (Fig. 1C, D), and this conformation allows for the formation of two salt bridges between the SXXS motif with phosphoserines and RXXR in the recognition helix. Analyses using the generalized-ensemble MD method revealed a conformational ensemble of molecular models of flexible IDR. The free-energy landscape of fuzzy complex can be unveiled in the atomic resolution. This technique can be applied to a variety of molecular systems and is expected to elucidate the molecular mechanisms of IDRs forming a fuzzy complex.

### 4.2 Metastable Multimer Assembly of IDRs

In contrast to the above example describing the multimodal interactions between IDR and an ordered region, multimer assembly of IDRs have also been reported. According to the amino acid sequence and condition, multimer assembly of IDRs can form diverse morphology from metastable aggregate (e.g., amyloid fiber) to dynamic fuzzy complexes (e.g., liquid droplet). For the former case, Amyloid β (Aβ) is a representative of proteins exhibiting fiber formation. Aβ forms periodic β-sheet structures, called cross-β structure, the element of which is short motif with hydrophobic sequence termed steric zipper [84]. Molecular mechanisms of oligomer formation with the steric zipper have been dissected with all-atom MD simulations. However, analysis of the free-energy landscape of higher-order complexes requires exploring a huge conformational space originated from the combination of many peptides. Most of the precedent all-atom MD studies for Aβ peptides focused on conformational ensembles of monomer or dimer of Aβ peptides. As an exception, Itoh and Okumura [85] analyzed the free-energy landscape of tetramer formation of Aβ29–42 fragment with the all-atom MD method. They applied the Hamiltonian replica permutation method combined with Suwa-Todo algorithm to enhance the conformational changes. The free-energy landscape indicated that Aβ29–42 fragment tetramer is incrementally formed by adding a monomer to another monomer or oligomer rather than through assembling two dimers. They discussed the importance of solvation entropy for this scenario.

### 4.3 Extreme Fuzzy Complex

In the fiber formation described above, IDRs form a metastable assembly, the structure of which is almost fixed in a long-time scale. Another class of IDR–IDR interactions form a dynamic fuzzy complex, the structure of which is frequently interconverted under thermal fluctuation. One prominent example of this is the formation of high-affinity complex between histone H1 and its nuclear chaperone prothymosin-α as reported by Borgia et al. [86] Integration of a variety of experimental techniques, such as NMR, FRET, and circular dichroism (CD) with a coarse-grained MD method has revealed that H1 and prothymosin-α interact with each other without forming stable conformations but with picomolar affinity. Their interactions are driven by the opposite net charges between these proteins. It is noteworthy that the force field applied in this coarse-grained simulation did not distinguish residues with the same charge as pointed out by Ruff et al. [87] Finer force fields may be required to dissect differences among residues. Another example is the 4.1G-CTD–NuMA complex reported by Wu et al. [88] They confirmed that these IDRs exhibited diverse conformations in bound state by using REMD simulation as well as single-molecule FRET and NMR experiments. The complex formation is driven by a pair of hydrophobic motifs and opposite charges in each IDR. Wang and Wang [89] termed this kind of dynamic IDR–IDR assembly as “extreme fuzzy complex”.

### 4.4 Liquid–Liquid Phase Separation (LLPS)

**Fig. 2:**
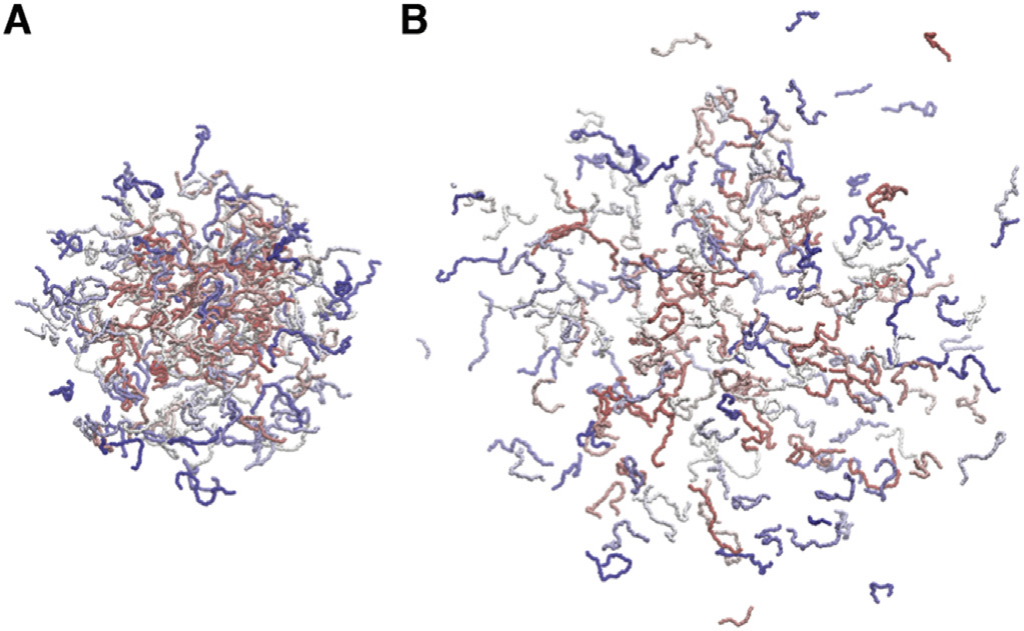
Liquid–liquid phase separation (LLPS). (A) A liquid droplet-like structure formed with polypeptides. Different chains are represented by different colors in gradation from blue to red. In this structure, many polypeptide chains gather loosely and fluctuate dynamically. (B) A dispersed structure of the same system. The liquid droplet-like structure disappears easily due to several factors (e.g., increase in temperature, post-translational modification). The figures are generated from coarse-grained MD simulations using myPresto/omegagene [92]. The system includes 200 chains each of which consists of 57 beads (one-bead per residue). Potential energy was calculated based on the hydrophobicity scale model [93] and Debye-Hückel approximation. The Langevin thermostat was applied. The details of this simulation will be published elsewhere.

Some IDRs form μm-sized molecular assemblies, or liquid droplets, without stable conformations (Fig. 2) [90] The protein droplets work as membraneless organelles with indispensable functions for a variety of biological processes that compartmentalize inside the cell by liquid–liquid phase separation (LLPS). For example, the RNA-binding protein FUS is a representative that forms a liquid droplet. A low-complexity (LC) IDR next to the RNA-binding globular domain is a keystone to forming the fuzzy assembly [91]. There are many phosphorylation sites within the IDR LC region and their modification controls the stability of the droplets by electrostatic repulsion.

Because the assembly is formed as a micrometer-order droplet, it is difficult to treat this system using the all-atom MD method with the explicit solvent model. MD studies for LLPS were conducted primarily by using a coarse-grained model with an implicit solvent model. Dignon et al. [[94], [95], [96]] have reproduced the liquid droplet formation using several proteins, including FUS, and the coarse-grained MD method. The simulations were conducted using various temperatures and the temperature-dependent behavior of the droplet formation was observed. The critical temperature was estimated based on the phase diagram. The results of their study indicated that inducing phospho-mimicking mutations destabilizes the liquid droplet, consistent with experimental observations, and the phosphorylation position in FUS does not affect significantly droplet stability. Das et al. [97] have applied a similar technique to investigate sequence determinants for LLPS based on a model sequence with various charge distributions. They have presented that the droplet is destabilized in sequences where charged residues are delocalized.

These recent trials using coarse-grained MD approaches with one-bead for one-residue successfully reproduced liquid droplet-like behavior of protein assemblies. Such molecular systems are difficult to observe using all-atom potentials and explicit solvent models with the current computational resources. At the same time, the details of the atomic interactions tend to be overlooked in the coarse-grained model. To accurately describe the molecular actions, the function forms and parameters would need to be adjusted. For instance, Dignon et al. [94] have described a model using a modified LJ function with its parameters, optimized to reproduce experimental *R*_g_ values for various IDRs. Further, the temperature dependence has been explicitly taken into account [98]. The CAMELOT method fits the parameters for reproducing the behavior of an all-atom model using machine learning techniques [99]. However, the physics of some specific interactions, such as cation–π interactions, considered to be key features of LLPS [100], are not explicitly included. It is expected that future studies will be on more fully developing the coarse-grained model.

## 5 Summary and Outlook

In this mini-review, the methodological issues and applications for IDR interactions of MD simulations are discussed. Simulations for IDRs have several difficulties: (i) force fields tailored for structured proteins are not suitable for IDRs (ii) large fractions of charged residues in IDRs would cause artifacts of electrostatic potentials originating from the PBC (iii) high conformational heterogeneity of IDRs makes exploration of the conformational space difficult. Advances in the methodology and computational power have tackled these problems. Some IDR-oriented force fields have successfully yielded conformational ensembles that agree with a variety of experimental observations. Recent studies have focused on unifying force fields for IDRs and those for structured regions [29]. Details of the PBC artifacts have been extensively evaluated, and non-Ewald methods may reduce the artifacts [40]. State-of-the-art enhanced sampling methods have revealed conformational ensembles including bound and unbound states for several IDR complexes using the all-atom explicit-solvent model [63,65,67,75,79,85]. Coarse-grained MD approaches have been developed to investigate higher-order assembly of IDRs, e.g., liquid droplet [94]. A recent trend of MD simulation studies for IDRs is a collaboration with experimental techniques such as CD, FRET, NMR, SAXS, and kinetic experiments. MD simulations provide atomic models explaining experimental results [74,86,88]. The results obtained from MD simulations suggest new experiments (e.g., design of mutants) [79,83]. Experimental data provide restrictions for MD simulations to narrow the conformational space to be explored [68]. Such an integrated approach promotes exceeding the limits of each approach alone.

As described above, recent studies have emphasized that a classification or a model describing molecular mechanisms should be considered as a spectrum for actual cases. Although two ideal models of coupled-folding and binding processes are presented, i.e., conformational selection and induced fit, many studies have suggested mixed scenarios between them [101]. With respect to the types of IDR interactions, the two categories focused in this mini-review, coupled-folding and binding and fuzzy complex, are not mutually exclusive. Protein complexes are considered to be on a spectrum between ordered complexes and extreme fuzzy complexes [11]. Protein structures should be considered on a spectrum from ordered to disordered. [102] Unified models of these spectra are needed for understanding the molecular mechanisms of protein interactions.

Toward this end, various molecular systems in the spectrum should be analyzed. The current landscape of IDR interactions analyzed by all-atom explicit-solvent MD simulations is limited to several representatives. To expand the view further, the applicability of MD simulations should be expanded via studies on the basics of the methodology, i.e., adjustments, evaluations, and benchmarks of a variety of simulation conditions including force field, and development of software. Although the force fields have been extensively developed, the current classical ones still fail to reproduce some of the details of molecular behavior [35,103,104]. Artifacts from various conditions including the PBC [40] and conditions for enhanced sampling [55] are not fully understood for IDR simulations. Because users must acquire some experience and technical knowledge to appropriately apply some kinds of simulation methods (e.g., enhanced sampling methods and coarse-grained simulations), development and maintenance of easy-to-use software are essential.

Because the various fundamental roles of IDRs in the physiology and diseases of the human body are known, dissecting their molecular details will provide great insights into therapies used. Moreover, unstructured peptides currently attract attention as candidates for new drugs. The MD method provides means of a structure-based drug design for unstructured drugs and their targets.

## Acknowledgements

K. K. was supported by JSPS KAKENHI Grant No. JP16K18526. J. H. was supported by JSPS KAKENHI Grant No. JP16K05517 and the Development of Core Technologies for Innovative Drug Development based upon IT from the Japan Agency for Medical Research and Development, AMED.
